# Navigating the Deluge: Unraveling the Impact of Floods in Kano Plains, Kenya

**DOI:** 10.4269/ajtmh.24-0216

**Published:** 2024-10-01

**Authors:** Stellah A. Chumbe, Bartholomew N. Ondigo

**Affiliations:** Laboratory of Infectious Diseases, Department of Biochemistry and Molecular Biology, Egerton University, Nakuru, Kenya

Sensing something amiss, I woke up at 4 am on a Wednesday to murmurs outside my home. As I stepped out of bed, my feet landed in a pool of freezing water, signaling the reality that floods had engulfed us. The previous night’s heavy showers and thunderstorms had hinted at the impending disaster, yet the full extent of what awaited us was unclear. It all began in October 2023, as forecasted heavy rains materialized. Despite early pleas from my village mates asking to be relocated to safer spaces before the onset of adverse rain or to construct gabions around the Nyando River to mitigate erosion, our appeals fell on deaf ears.

My family’s home lies in the Kano Plains, nestled in the heart of western Kenya, characterized by undulating landscapes and a series of fault line escarpments that descend onto the plains. The region’s fertile clay soil has supported our livelihood through the growth of crops and livestock. Although the vast tracts of land are a blessing, they have also become a curse over time because of their vulnerability to floods, which bring forth new waves of challenges and devastation, turning into a recurring nightmare for us.

The loud voices from the neighbors confirmed that the calamity was not isolated to our household. Our legs submerged in cold water, my siblings and I tried to remove the water using basins and containers from the house, pouring it out until 6 am. However, the persistent ongoing drizzle and relentless return of the poured-out water rendered our efforts futile.

As a native of this land, I have witnessed the cyclical nature of this natural disaster, with each passing year bringing a new wave of challenges and destruction. But none of that prepared me for the moment when most of our belongings were swept away, leaving us with only the clothes on our backs and a profound sense of helplessness. We had to walk in the water, which reached above our knees. The floodwaters did not just steal our possessions. The deluge bore down on our house, where we had lived for years, cracking the muddy walls. The heavy winds blew away one side of the roof. The sofas were soaked in water, with no dry area left for us to step on. All the history and memories we held dear, the sense of security we once knew, were lost.

As the sun rose, it became evident that the floodwaters not only devastated the newly planted rice fields but also transformed vast areas into muddy wastelands and rendered the roads impassable. The hard-planted crops, a testament to our labor, were swept away like dreams carried off by the current, raising fears of crop failure. The sight of our cattle, sign of our wealth, added to our heartache. Some cattle were mooing for help, whereas others had been struck down by lightning. We could hear people weeping. Loss and despair gripped the community as lives were upended.

With our home partially submerged and no other option, we made the difficult decision to seek refuge at a rescue center. My family and I found ourselves taking shelter at Rabuor Secondary School, one of the evacuation centers amidst the unfolding crisis. I followed my parents as we surveyed the classroom that was to serve as our bedroom. There were five classrooms, each seeming to hold a capacity of 30 students; but with all seeking refuge here, approximately 500 people crowded into the place. We stayed in the classrooms as the rains continued to fall, penetrating through the holes in the roof and the broken windows. The cold wind swept through the broken doors and whipped past the electricity-less bulbs on the roof trusses. As I stared at the faces of the children, old men, and women in the camp, any semblance of happiness fled from our quarters.

Night fell. Some of us used our phone’s flashlight and uncertain where we could charge the phones or what we would use the next night. Others relied on the moonlight shining through the rooftop and broken windows. We had to squeeze ourselves so as to sleep, not minding gender and age differences. My mother spread her wrapper on the floor, and with my young sister, we huddled together on it for warmth and comfort amidst the uncertainty. As I watched my father, lost in deep thoughts, perhaps thinking about what the future held, my mother asked me not to worry. But I could not stop the thoughts running in my mind. She reassured me that once the floods subsided, we would return home. However, I could not help but wonder: How will we manage? What will become of our homes? All our animals and crops are gone. The trauma of displacement weighed heavily on my young shoulders. Despite being sheltered in a classroom, it seemed to me as though our days of attending school were over. That night, sleep eluded me as mosquitoes incessantly buzzed around my ears and the cold air came in through the broken windows, making conditions unbearable.

The media reported that the bursting banks of the Nyando River displaced families, who were sheltering at the Ahero Shopping Center as well as those huddling with us. Everyone who crammed in the shelters faced the specter of waterborne diseases and increased mortality rates, compounding an already difficult situation. Hunger loomed large, exacerbating the already dire situation we were facing.

It was about 36 hours after losing our home, while we were still in the school, that the media personnel came. As expected, their interest was in capturing firsthand accounts of the situation. Ronald Okumu, 45, one of the men in our camp whose home was completely submerged in water and who was struggling with a headache and cold symptoms, lamented on camera of the challenges in accessing healthcare due to the distance to the nearest health facility. The hospital that usually serves the community was submerged in floods and hence was not operational. Margaret Abala, 27, was breastfeeding her 6-month-old baby while tears streamed down her face as she was interviewed. She was hungry and worried that her breast milk supply would not be enough.

We were left at the mercy of nongovernmental organizations—the Kenya Red Cross and the Kibos Sugar Company—for humanitarian and medical support, including donated blankets, utensils, mosquito nets, and food rations. Margaret and other breastfeeding mothers, in addition, were provided with porridge flour and some milk for their babies. Those feeling unwell received medication, yet the question still lingering in my mind was “How sustainable are these short-term solutions?” Unfortunately, the food supply fell short within a week, depriving us of the luxury of at least two meals a day. The babies kept wailing, possibly owing to hunger and scarcity of clean drinking water. As the eldest sibling in our family of five, most of the time I had to take it upon myself to relinquish part of my meals to my little siblings, aged 12 and 8 years, in a bid to provide them with sufficient feeding. It was a painful sacrifice, but in the face of adversity, we had to support each other as best we could.

Because of the scarcity of clean drinking water caused by the contamination of wells from the floodwaters, a cholera outbreak added to our distress. It all began when my cousin started experiencing severe diarrhea, followed by Margaret and other young children, who were wailing and vomiting. They had to be rushed to Ahero Hospital, whereas others were referred to Jaramogi Oginga Odinga Teaching and Referral Hospital. In the hospital, I felt utterly confused and helpless as I watched my cousin suffer mercilessly because of the scarcity of drugs and beds for patients. He eventually died in my mother’s arms. It was a heart-wrenching sight, and I could not help but curse the flood calamity for inflicting such pain on us. Not knowing where or when we would lay him to rest, we left him in the morgue. Tears never stopped cascading down my cheeks.

The severity of the situation in the hospital prompted leaders to visit the camps, including ours. However, their response fell short of addressing the urgent needs. It was disheartening to witness leaders arriving with milk and bread in trucks while people were suffering. We needed tangible solutions: larger rescue centers to accommodate the growing numbers of displaced families, medicine to treat the sick, clothes, or even a promise to allocate us land somewhere to build temporary houses.

As I moved to a quiet corner of the camp and observed people taking the milk and bread because they were hungry, my mind swirled with thoughts. The challenges posed by recurring floods demand a multifaceted response, one that encompasses not only immediate relief but also sustainable, long-term solutions. The safety and well-being of individuals, such as Margaret’s baby and others, hang in the balance. But amidst the uncertainty, there is hope.

This is my story. I, Chumbe A. Stellah, wrote this story with help, guidance, and encouragement from Bartholomew N. Ondigo.

